# Building sustainable TB care systems: managing incentives in private sector engagement

**DOI:** 10.5588/ijtldopen.25.0340

**Published:** 2025-11-12

**Authors:** L. Kimbo, A. Rashid, E. Wandwalo, E.O. Masini, G. Stallworthy, P. Heitkamp, M.A. Yassin

**Affiliations:** 1 Global Fund to Fight AIDS, Tuberculosis and Malaria, Geneva, Switzerland;; 2 Independent consultant, Dar es Salaam, Tanzania;; 3 The Bill and Melinda Gates Foundation, Seattle, USA;; 4 McGill International TB Centre, TBPPM Learning Network, Montreal, Canada.

**Keywords:** tuberculosis, financial incentives, non-financial incentives, sustainability, innovative strategies

## Abstract

Private sector engagement (PSE) plays a vital role in enhancing TB care. Global Fund-supported PSE activities contributed to improved TB notifications and treatment outcomes, especially where non-financial incentives were complemented by financial incentives, delivered directly or via social health insurance. Transitioning such activities to government-led health systems is essential for long-term sustainability and to end TB. Well-designed incentive strategies could ensure quality care where patients seek services. However, payment delays, administrative burdens, and donor dependency threaten sustainability. Governments need to address these challenges to transition programs into people-centered, integrated primary healthcare systems, ensuring access to training, diagnostics and program drugs.

TB remains a significant global public health challenge, and the leading infectious diseases killer. Despite being both preventable and curable, an estimated one-quarter of the global population is infected with TB bacteria, 10.8 million developed TB disease in 2023, and 1.25 million died from the disease.^1^ Additionally, 8.2 million people with TB were notified to national authorities, with over two-thirds of cases concentrated in eight countries: India, Indonesia, China, the Philippines, Pakistan, Nigeria, Bangladesh, and the Democratic Republic of the Congo.^2^ Recognizing the pivotal role of the private sector in the TB response, particularly in high-burden countries where a significant proportion of the population seeks healthcare, enhancing PSE is critical. The extensive involvement of private healthcare providers (PHPs) presents a significant opportunity to expand and improve TB service coverage and quality. The WHO End TB Strategy^3^ and the Global Fund Strategy for 2023-2028^4^ emphasize strengthening PSE to provide accessible, affordable, and high-quality services integrated with public health systems. This commentary examines the influence of both financial and non-financial incentives in improving TB case notifications within the private sector, exploring key enablers, governance improvements, challenges and policy considerations, particularly within UHC efforts in LMICs. The challenges associated with incentive processes are shown in the Figure.

**Figure.**
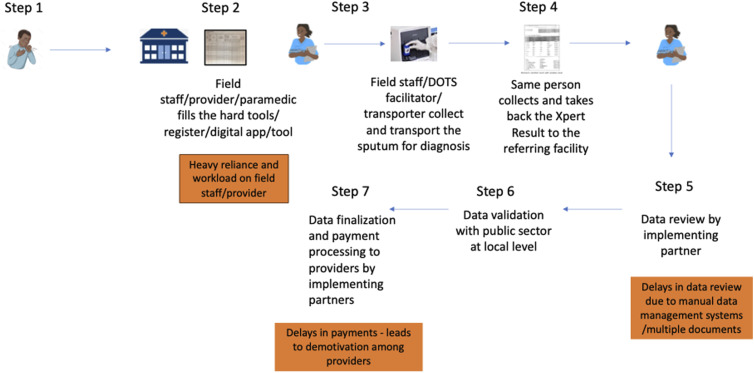
Challenges associated with incentive processes.

## WHY THE PRIVATE SECTOR?

PHPs play a significant role in seven high-burden TB countries (India, Indonesia, Philippines, Pakistan, Nigeria, Bangladesh, Myanmar) where approximately 75% of the population initially seeks healthcare from private providers.^5^ However, due to weak engagement of these providers, private for-profit notifications account for only 35% of total notifications, ranging between 23% and 42%.^6^ Leveraging the private sector in TB care and management efforts is key to delivering comprehensive TB care services close to patients’ homes and at times convenient to them, thereby reducing out-of-pocket costs and improving cost-effectiveness. Many more patients are diagnosed and treated for TB in the private sector than official records reflect, or they may visit multiple healthcare providers before reaching the public sector. A patient pathway analysis across five countries revealed that 66% of patients initially sought care in private facilities, which had significantly lower TB diagnostic capacity compared to public facilities.^7^ This highlights the need to integrate PSE at the primary healthcare level, where most patients seek care. This can improve care quality, increase efficiency, and enhance access to TB services. This approach reduces unnecessary referrals to higher-level facilities, promotes early diagnosis and treatment, and supports sustainable TB financing.

## INSIGHTS INTO PSE: EVIDENCE, GAPS AND THE WAY FORWARD

India successfully transitioned private provider engagement for TB from donor-funded projects to a government managed fully-funded model outsourced to private agencies.^6^ Through government provisioning, private providers receive US$ 5.73 per notified TB patient and a further US$5.73 per successful treatment. In the first three quarters of 2023, US$3.4m was paid to 36,810 private providers.^8^ We assessed Global Fund’s investments in PSE across six high-burden countries: Bangladesh, Indonesia, Nigeria, Pakistan, the Philippines, and Tanzania. An in-depth analysis of financial and non-financial incentives for PHPs was conducted in Nigeria, Pakistan, and the Philippines. The assessment integrates insights primarily gathered from online or face-to-face key informant interviews with key stakeholders, including National TB programs, country implementing and funding partners, the Global Fund’s country teams, PHPs, and field visits. The data from the WHO PPM dashboard 2024,^9^ and published literature, provide a comprehensive overview of the private sector TB care landscape. Findings show that in Nigeria, Pakistan, and the Philippines, PHPs financial incentives range from $0.13 and $13.34 across the TB care cascade (Table). The incentives are context specific and the variation in average incentive per patient diagnosed and successfully treated across countries reflects differences in service delivery models, the scope of incentivized activities, and provider engagement strategies. For example, Nigeria's model covers a broader range of services across the TB care cascade, resulting in higher cumulative incentives. In contrast, Pakistan and the Philippines follow more focused approaches, targeting fewer service points and involving fewer provider types. These differences highlight the influence of country-specific program designs, operational priorities, and donor funding levels and cost-effectiveness of the incentive models implemented.

**Table.** Overview of types of financial incentives across the TB care cascade (2021-2023).

Type of provider	Service for which incentives are provided	Incentive amount (US$)	Total count of the service	Total number of providers performing the service	Average number of services per provider per year	Average incentive per provider per service per year (US$)
**Nigeria**						
Patent medicine vendor (PMV)**^A^**	Referral	1.22	577,252	6,605	29	36
PMVs	Confirmed TB case from referral	12.17	53,034	2,565	7	84
Clinician/Medical Officer	Treatment initiation	7.30	30,860	1,905	5	39
Radiographer	Chest Xray	14.60	30,275	499	69	295
Hub (PFP, FBO)	Treatment monitoring	7.30	58,726	1,905	10	75
Hub (PFP, FBO)	Treatment success with AFB follow-up	0.49	75,505	1,905	13	6.5
Linkage Coordinators	Client linkage	1.22	577,252	430	447	546
Average total incentive per patient diagnosed and successfully treated (referral, confirmed TB case, treatment initiation, chest Xray, treatment monitoring, treatment success, client linkage) $51.62						
**Pakistan**						
GP	Case notification with treatment outcome	2.98	414,537	8,610	16	48
Lab technician	AFB slide	1.0	1,910,676	650	979	979
Hospital	Xpert testing (monthly operational cost)	∼90		94 hospitals		
Average total incentive per patient diagnosed and successfully treated (microscopy, case notification and treatment outcome) $26.98						
**Philippines**						
Private physicians	Notification of TB patient tested with mWDR	4.45	24,867	1,587	5	23
	Treatment outcome	13.34	9,980	388	8.5	112
RX Pass sites	mWDR test	30		103		
Average total incentive per patient diagnosed with mWDR and successfully treated is $17.79						

A The total incentive is capped at 7 presumptive referrals per diagnosed TB case.

PFP = private for profit; FBO = faith based organization

A review of 2021-2023 data from three countries revealed significant increases in TB notifications and treatment initiation in the private sector, driven by Global Fund-supported programs that combine financial and non-financial incentives including diagnostics access, supervision, monitoring, training, provider support and data tracking. Nigeria reported an 86% increase in TB notifications, with private providers contributing 29% of notifications. Pakistan had a 68% increase, with 45% coming from the private sector, while the Philippines saw a 100% surge in notifications, with the private providers contributing 24% in 2023.^6,2^ Nigeria offers the highest incentive per diagnosed and treated TB case ($52), covering 20% of total and 72% of private notifications. Pakistan follows ($27) for 31% of total and 72% of private notifications, while the Philippines ($18) covers 24% and 97%, respectively. Total incentives paid (2021–2023): Nigeria ($3.26M), Pakistan ($1.19M), Philippines ($240K), excluding overhead and support costs. Despite the existence of more than 100,000 private sector facilities across these countries, engaging 5,506 facilities in Nigeria, 13,000 in Pakistan and 14,112 in the Philippines^9^ still yielded high notification rates. An overview of types of financial incentives across the TB care cascade (2021-2023) is presented in the Table.

Simplified, automated incentive systems geared towards payment for results, effectively support private sector providers, informal providers, laboratory and other field staff, without adding administrative burdens. Such schemes incentivize quality care and adherence to treatment guidelines e.g. Pakistan employs a straightforward system with two payments: $2.98 for treatment initiation and outcome and $1 for laboratory testing. Payments are validated through quarterly validation of referral forms, updated upon treatment completion. Similarly, the Philippines offers $4.45 for a confirmed molecular WHO-recommended diagnostic test (mWRD) and an additional $13.34 for a documented treatment outcome, aligned with the PhilHealth insurance program and supporting future transition to public funding. Nigeria’s incentive model effectively engages private providers^10^ and costs $51.62 per patient. Payments span screening, diagnosis, and treatment but face delays due to manual data entry, Excel tracking, and multi-level verification, impacting efficiency and provider engagement. In 2014, Indonesia launched its national social health insurance scheme, JKN (administered by BPJS), which marked a major milestone in healthcare reform and now includes embedded incentives for TB services. The insurance covers both public and private healthcare providers. This initiative played a crucial role in integrating TB notification into the broader health system by aligning insurance payment mechanisms with TB case reporting, rather than being paid directly to private providers. Since 2023, BPJS began requiring TB notification as a condition for paying hospital TB claims, increasing notifications from 93,248 (2021) to 235,706 (2023). Private primary care notifications rose from 3,071 (2021) to 12,370 (2023) but remain low (at just 2%) prompting JKN payments reforms to enhance engagement, testing access and drug availability. Successful engagement of private primary care providers, commensurate with their role in patients’ early care-seeking, will depend on the successful implementation of these reforms.

## DISCUSSION

A sustainable, government-led TB response, that fully engages the private sector, requires provider support beyond diagnosis, ensuring management of patients throughout treatment. This calls for a holistic support system combining both financial and non-financial incentives through the TB care cascade. It includes providing free or subsidized diagnostics and government-supplied TB medicines for timely detection, standardized treatment, and reduced financial burden on patients and private providers. Non-financial incentives, such as accreditation, professional networks, and training, enhance provider engagement and care quality. Effective case-based data systems facilitate program efficiency and accountability. Joint supervision and policy alignment also foster trust. This comprehensive approach ensures private providers remain committed partners in TB control efforts.

For the countries analyzed, financial incentives and enablers for private providers have typically been funded by donors, notably the Global Fund and USAID, with India being the only country to integrate them into domestic government funding at scale, including through successful transition of the Global Fund-funded PPSA (patient-provider support agency) model to domestic funding and further scale up. Sustainability requires integrating these interventions into governments’ strategic health purchasing programs, using digital systems to ensure efficiency and accountability. Countries with comprehensive social health insurance programs, including primary care, may leverage them to optimize TB incentives and enablers, while others (such as India) may develop separate arrangements for TB-specific purchasing. The full integration of private providers into routine NTP’s activities remains challenging due to weak regulatory systems, fragmented service delivery, limited data integration, and a lack of sustainable financing mechanisms. The Global Fund has played a catalytic role in this transition by supporting scalable PSE models and integrating them into national strategies as for example done in India. Nonetheless, many PSE efforts remain project-based and externally funded, underscoring the need for stronger domestic financing and institutionalization through mechanisms such as UHC schemes, insurance and routine budget allocations.

## CONCLUSION

The private sector’s resilience during crises, such as COVID-19, underscores its role in health system strengthening.^11^ As global aid shifts and external funding shrinks, increasing domestic financing, integration of TB into the national health budget, use of health insurance and government stewardship are crucial. Integrating private providers into strategic health purchasing systems ensures sustainability, advances UHC, and supports the achievement of end TB goals.
